# Vibrio cholerae Alkalizes Its Environment via Citrate Metabolism to Inhibit Enteric Growth *In Vitro*

**DOI:** 10.1128/spectrum.04917-22

**Published:** 2023-03-14

**Authors:** Benjamin Kostiuk, Mark E. Becker, Candice N. Churaman, Joshua J. Black, Shelley M. Payne, Stefan Pukatzki, Benjamin J. Koestler

**Affiliations:** a Department of Medical Microbiology and Immunology, 6-020 Katz Group Centre, University of Alberta, Edmonton, Alberta, Canada; b Department of Cell and Developmental Biology, Northwestern University Feinberg School of Medicine, Chicago, Illinois, USA; c Department of Biological Sciences, Western Michigan University, Kalamazoo, Michigan, USA; d Department of Molecular Biology and Genetics, Johns Hopkins University School of Medicine, Baltimore, Maryland, USA; e Department of Molecular Biosciences, The University of Texas at Austin, Austin, Texas, USA; f Institute for Cellular and Molecular Biology, The University of Texas at Austin, Austin, Texas, USA; g Department of Biology, The City College of New York, New York, New York, USA; University of Washington

**Keywords:** *Vibrio cholerae*, metabolism, citrate, citrate lyase, oxaloacetate decarboxylase, carbonate, enteric bacteria, pH

## Abstract

Vibrio cholerae is a Gram-negative pathogen, living in constant competition with other bacteria in marine environments and during human infection. One competitive advantage of V. cholerae is the ability to metabolize diverse carbon sources, such as chitin and citrate. We observed that when some V. cholerae strains were grown on a medium with citrate, the medium’s chemical composition turned into a hostile alkaline environment for Gram-negative bacteria, such as Escherichia coli and Shigella flexneri. We found that although the ability to exclude competing bacteria was not contingent on exogenous citrate, V. cholerae C6706 citrate metabolism mutants Δ*oadA*-1, Δ*citE*, and Δ*citF* were not able to inhibit S. flexneri or E. coli growth. Lastly, we demonstrated that while the V. cholerae C6706-mediated increased medium pH was necessary for the enteric exclusion phenotype, secondary metabolites, such as bicarbonate (protonated to carbonate in the raised pH) from the metabolism of citrate, enhanced the ability to inhibit the growth of E. coli. These data provide a novel example of how V. cholerae outcompetes other Gram-negative bacteria.

**IMPORTANCE** Vibrio cholerae must compete with other bacteria in order to cause disease. Here, we show that V. cholerae creates an alkaline environment, which is able to inhibit the growth of other enteric bacteria. We demonstrate that V. cholerae environmental alkalization is linked to the capacity of the bacteria to metabolize citrate. This behavior could potentially contribute to V. cholerae’s ability to colonize the human intestine.

## INTRODUCTION

For bacteria to thrive in a competitive environment, they must be highly effective in resource acquisition to proliferate their niche (1, 2). Bacteria employ both passive and active forms of competition (2–4). Active processes include the secretion of toxins or the sequestration of resources (1, 3). Passive mechanisms include the secretion of waste products of their secondary metabolic pathways, making the environment hostile for their competitors; such secondary metabolites are not required for the producing organism and are referred to as allelochemicals (5).

Vibrio cholerae, the causative agent of the diarrheal disease cholera, is a Gram-negative pathogen that resides primarily in marine reservoirs and causes disease upon human ingestion (6). Because the pathogenic cycle of V. cholerae involves transitioning between its natural marine environment and the human host, V. cholerae has evolved to be highly competitive in both of these environments (6). During these transitions, V. cholerae interacts with many different types of microbial organisms, including various eukaryotes and bacteria of the same or other species (7, 8). As a consequence of residing in diverse microbial communities, V. cholerae has evolved multiple competitive mechanisms that are effective against other members of its species, other bacterial species, or eukaryotic predators (1, 3, 7).

One such V. cholerae survival mechanism is using multiple carbon sources for energy (9). A prominent example is the ability of V. cholerae to use chitin as a carbon source, as chitin is abundant in marine environments (10, 11). In addition to chitin, V. cholerae can metabolize other carbon sources, such as dietary citrate, that some of its competitors cannot utilize (12). Citrate metabolism is widely conserved among V. cholerae strains (13, 14) and contributes to competitiveness in a V. cholerae infant mouse model of infection (15). Strains of V. cholerae that successfully colonize humans also endure the low pH stress of the stomach and small intestine (9, 16). The capability of V. cholerae to thrive in multiple environments has given rise to novel mechanisms of competition. For example, V. cholerae actively secretes vibriobactin, and this siderophore sequesters iron to provide this essential nutrient for itself and to prevent other species from using it (1). Other examples include osmotolerance (17), resistance to bile acids (18–20), and biofilm formation.

V. cholerae is responsible for seven recorded pandemics. Pandemic strains are divided into two biotypes, namely, the seventh pandemic V. cholerae O1 El Tor biotype and the sixth pandemic O1 classical biotype, which evolved as distinct lineages (21–25). Differences in V. cholerae metabolism profiles are implicated in significant differences in intraspecies fitness. Notably, some El Tor strains produce 2,3-butanediol when metabolizing glucose; this metabolism creates a more favorable environment for the survival of El Tor strains than classical V. cholerae pandemic strains, which generate an unsuitably low pH when grown in the presence of glucose (25, 26). Here, we investigated whether V. cholerae C6706, an El Tor strain, uses its ability to metabolize citrate for growth advantages, and we present a model for a competition mechanism in which V. cholerae C6706 defines its chemical microenvironment through the metabolism of citrate. Using a simple *in vitro* assay, we show that V. cholerae C6706 metabolites create an environment hostile to competing bacteria through the increase in pH and bicarbonate production.

## RESULTS

### V. cholerae C6706 inhibits the growth of enteric bacteria.

The V. cholerae El Tor strain C6706, isolated during the ongoing 7th pandemic (27), suppresses the growth of other microorganisms, such as Escherichia coli and *Shigella* spp., in a cross-streaking assay on citrate-containing media when grown for an extended time (54 h) (28). A cross-streaking assay involves growing a streak of V. cholerae down the center of an agar plate. Following bacterial growth, bacteria are scraped off. The plate is subjected to chloroform vapor to remove residual bacteria, and a single line of an indicator strain like E. coli is streaked perpendicular to the original bacterial growth. The plates are incubated overnight, and the growth of the indicator strain is recorded. A streak of V. cholerae C6706 grown on nutrient broth agar supplemented with citrate (CB), but not on nutrient broth agar, resulted in inhibition of the growth of E. coli or *Shigella* spp. (29). E. coli or *Shigella* spp. did not inhibit V. cholerae growth (29). Because V. cholerae is no longer present on the agar plate when E. coli inhibition occurs, the interpretation was that growth on citrate stimulated V. cholerae secretion of an unknown factor.

**FIG 1:**
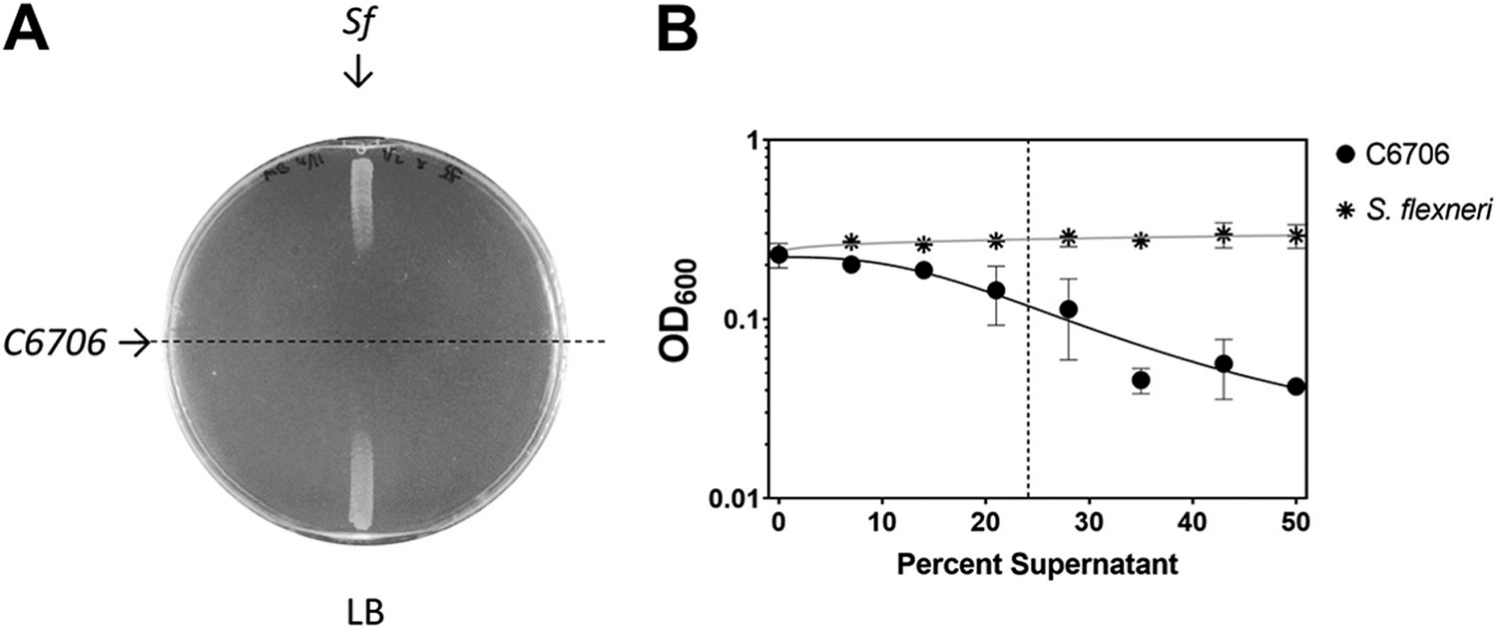
Extended culture of V. cholerae C6706 inhibits enteric growth. (A) A cross-streak assay demonstrates that V. cholerae C6706 inhibits the growth of S. flexneri CFS100. V. cholerae C6706 was grown for 72 h on LB agar. Bacteria were scraped off the plate, followed by a chloroform vapor treatment to kill residual cells. S. flexneri CFS100 was then streaked perpendicular to the V. cholerae growth and incubated for approximately 12 h. There was a clear zone of inhibition proximal to where the V. cholerae was originally located. (B) The supernatant inhibition assay demonstrates that V. cholerae cell-free supernatants inhibit S. flexneri CFS100 growth. V. cholerae C6706 or S. flexneri CFS100 was grown in LB for 72 h. Cultures were then centrifuged, supernatants were collected, and supernatants were filtered through a 0.022-μm PVDF filter. Supernatants were then diluted in LB at different ratios. The ability of S. flexneri CFS100 to grow in these supernatants was determined by measuring the OD_600_ after 6 h. V. cholerae supernatants inhibited S. flexneri growth in a dose-dependent manner, whereas S. flexneri supernatants had no inhibitory effects on S. flexneri growth. The IC_50_ (indicated by the dotted line) of V. cholerae supernatants was calculated to be 24.1%. Each point shows the mean of 3 replicates, and error bars show standard deviation. Trendlines show a nonlinear regression (log inhibitor versus response, four parameters).

Two groups had proposed previously that this behavior is competitive where an unidentified bacteriocin-like compound with bactericidal activity against E. coli is secreted (28, 30). A third group suggested a metabolic by-product was responsible for this growth inhibition (29). We confirmed this phenotype and found that prolonged growth (48 h) of V. cholerae C6706 on lysogeny broth (LB) agar before cross-streaking also resulted in growth inhibition of the closely related enteric bacterium S. flexneri, suggesting that the effect was stimulated by, but not dependent on, citrate present in the media for V. cholerae C6706 (Fig. 1A). We also performed a supernatant inhibition assay, where V. cholerae C6706 supernatants were centrifuged and filter sterilized to remove bacteria and debris, and then we measured the ability of the supernatant to prevent E. coli or S. flexneri growth. This process allowed us to quantitatively compare the inhibition properties of V. cholerae C6706 by calculating the half-maximal inhibitory concentration (IC_50_). We found that after growth in LB, V. cholerae C6706 cell-free supernatants had dose-dependent inhibitory activity against S. flexneri, whereas S. flexneri cell-free supernatants did not impede the growth of S. flexneri (Fig. 1B).

**FIG 2:**
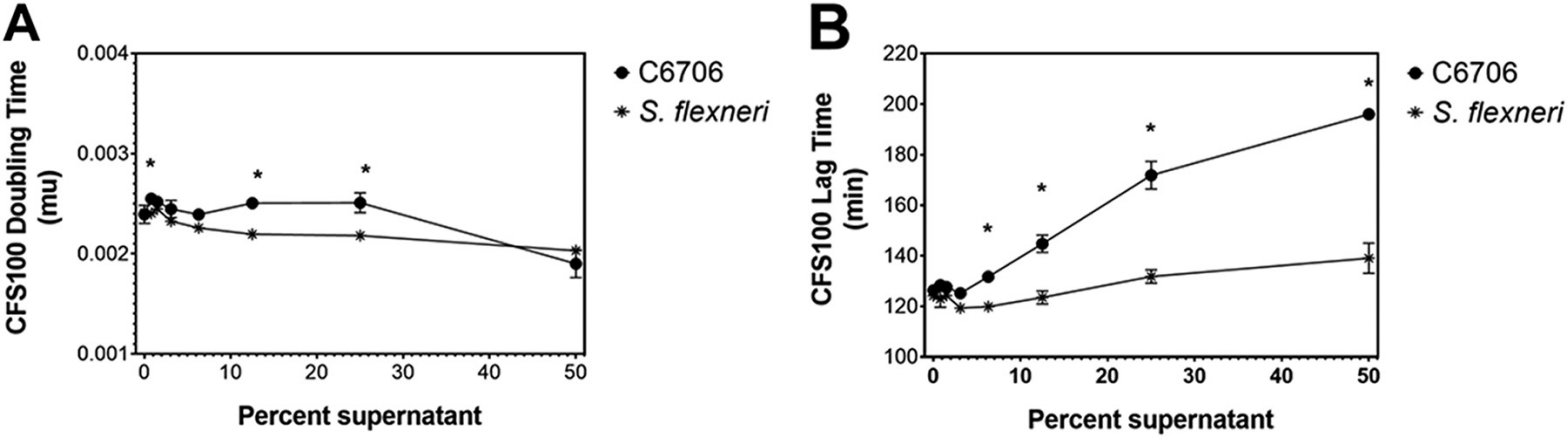
Effect of V. cholerae C6706 supernatants on S. flexneri growth. The supernatant inhibition assay was performed using supernatants derived from V. cholerae C6706 or S. flexneri CFS100, and the growth of S. flexneri CFS100 was monitored by OD_600_ measurements. The growth rate and lag time of S. flexneri CFS100 grown in different concentrations of supernatant derived from V. cholerae C6706 or S. flexneri CFS100 was quantified using the R grofit package (68). Each growth curve was replicated in triplicate. (A) We observed a modest increase in doubling time caused by the V. cholerae C6706 supernatant, relative to the supernatant derived from S. flexneri CFS100. (B) We observed a significant, dose-dependent increase in lag time in S. flexneri CFS100 growth caused by V. cholerae C6706 supernatants, relative to the S. flexneri CFS100 supernatants.

We sought to determine if the inhibitory effect of V. cholerae C6706 supernatants on S. flexneri growth in liquid media was bacteriostatic or bactericidal in nature. We grew S. flexneri in various concentrations of supernatant derived from the V. cholerae C6706 strain and S. flexneri and measured growth (optical density at 600 nm [OD_600_]) over time. We then determined the CFS100 growth rate and lag times under each of these conditions. We observed a modest effect on the growth rate of S. flexneri when it was grown in various concentrations of supernatant from either strain (Fig. 2A); however, there was a significant dose-dependent increase in the lag time prior to S. flexneri growth in V. cholerae C6706 supernatant, relative to S. flexneri grown in equivalent concentrations of S. flexneri supernatant (Fig. 2B), suggesting that this inhibitory effect is largely bacteriostatic in nature.

### Citrate metabolism contributes to V. cholerae C6706 inhibition of enteric bacteria.

To determine if the citrate-stimulated competition mechanism of the El Tor strain C6706 is a common trait of V. cholerae, we investigated whether other strains share this phenotype. We first determined the ability of 15 V. cholerae strains to grow on citrate using Simmons’ citrate agar (31). This test relies on an organism to grow using citrate as a sole carbon source. The strains used in this study included both environmental and pandemic-causing strains (32). Surprisingly, we found heterogeneity not only in the ability of various V. cholerae strains to create a hostile environment but also on the ability of V. cholerae strains to grow using citrate. While all strains were able to grow in LB or CB, we found that 9 of the 15 V. cholerae strains could grow on Simmons’ citrate agar (Table 1). Of those 9 strains, only five were able to create a hostile environment for E. coli in a cross-streak assay (Table 1). V. cholerae C6706 was able to grow in its own conditioned media. Some V. cholerae strains (Table 1) grew on citrate provided as the sole carbon source and yet were not able to inhibit the growth of E. coli. This finding is consistent with a previous study that noted no correlation between V. cholerae citrate metabolism and enteric inhibition from clinical V. cholerae isolates (30). Likewise, we did not observe any notable correlation between the source of the V. cholerae strains (i.e., pandemic or environmental) and the ability to create a hostile growth environment for E. coli (32).

**TABLE 1:** V. cholerae strains differ in their ability to metabolize citrate and prevent E. coli growth in a cross-streaking assay

V. cholerae producing strain^*a*^	Results of cross-streak assay			
	Growth on Simmons’ agar	pH of liquid LB medium, 48 h^*b*^	Growth of E. coli MG1655	Growth of V. cholerae C6706
C6706	+	9.0 ± 0.2	−	+
C6706 Δ*citE*::Tn	+	n.d.	+	+
C6706 Δ*citF*::Tn	+	8.1 ± 1.1	+	+
N16961	+	7.5 ± 1.2	−	+
O395	+	6.7 ± 0.1*	−	+
DL4211	+	9.1 ± 0.2	−	+
1587	+	7.6 ± 1.2	−	+
V52	+	7.5 ± 1.2	+	+
MZO-3	+	6.7 ± 0.0*	+	+
27-4080	+	7.6 ± 1.0	+	+
MAK-757	+	7.2 ± 0.1*	+	+
AM19226	−	7.3 ± 0.0	+	+
V51	−	7.6 ± 1.0	+	+
NIH41	−	6.9 ± 0.0*	+	+
MZ02	−	7.1 ± 0.0*	+	+
C6709	−	7.6 ± 1.0	+	+
CA401	−	7.0 ± 0.0*	+	+

a Fifteen V. cholerae strains analyzed in this study can be grouped into three distinct groups based on their ability to metabolize citrate as well as their ability to prevent the growth of E. coli in a cross-streaking assay on CB. The strains with no shading are able to grow on Simmons’ citrate agar and prevent E. coli growth. The light-gray-shaded strains are able to grow on Simmons’ citrate agar but not prevent the growth of E. coli. Finally, the dark-gray-shaded strains are not able to grow on Simmons’ citrate agar and subsequently are not able to prevent the growth of E. coli. Analysis was replicated at least three times.

b For pH values, * indicates statistical significance compared with C6706, as determined by one-way ANOVA with Dunnett’s posttest (*P* < 0.05); one outlier was identified and removed using the ROUT test (C6706, pH 6.8). n.d., not determined.

**FIG 3:**
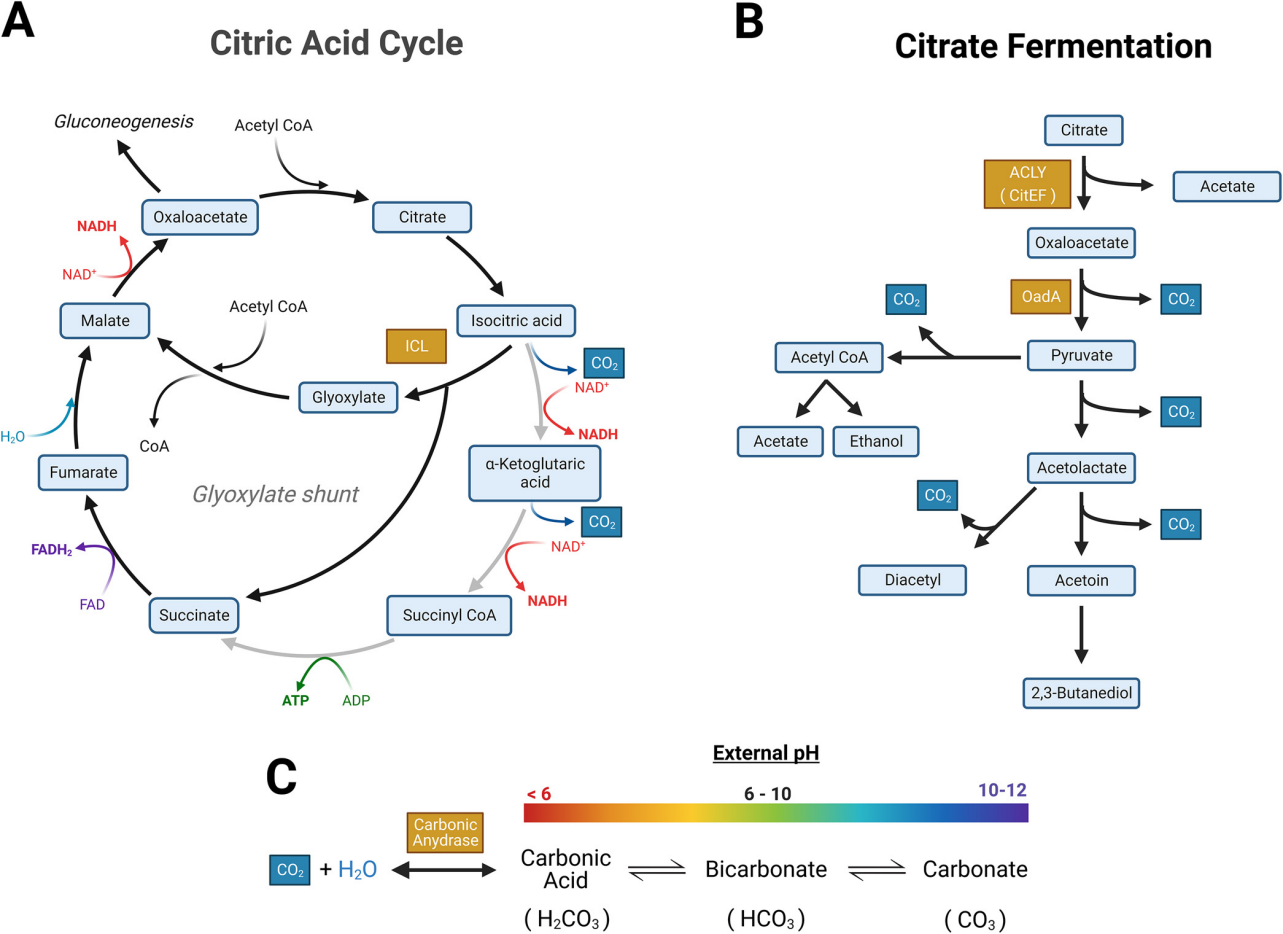
Conceptual model summarizing that the metabolism of citrate under basic conditions favors the formation of carbonate. (A) Diagram of the citric acid cycle in V. cholerae, highlighting the glyoxylate shunt. (B) Abridged metabolic flow diagram showing the fermentation of citrate to 2,3-butanediol or acetyl-CoA, producing CO_2_ as a by-product (in blue). (C) CO_2_ is converted to carbonic acid through the enzyme carbonic anhydrase. An equilibrium exists between carbonic acid, bicarbonate, and carbonate. More basic conditions favor the production of the negatively charged carbonate. Enzymes of interest in this study are highlighted in orange. Other enzymes and other cofactors have been omitted to emphasize the production of CO_2_. Created with BioRender.com.

**FIG 4:**
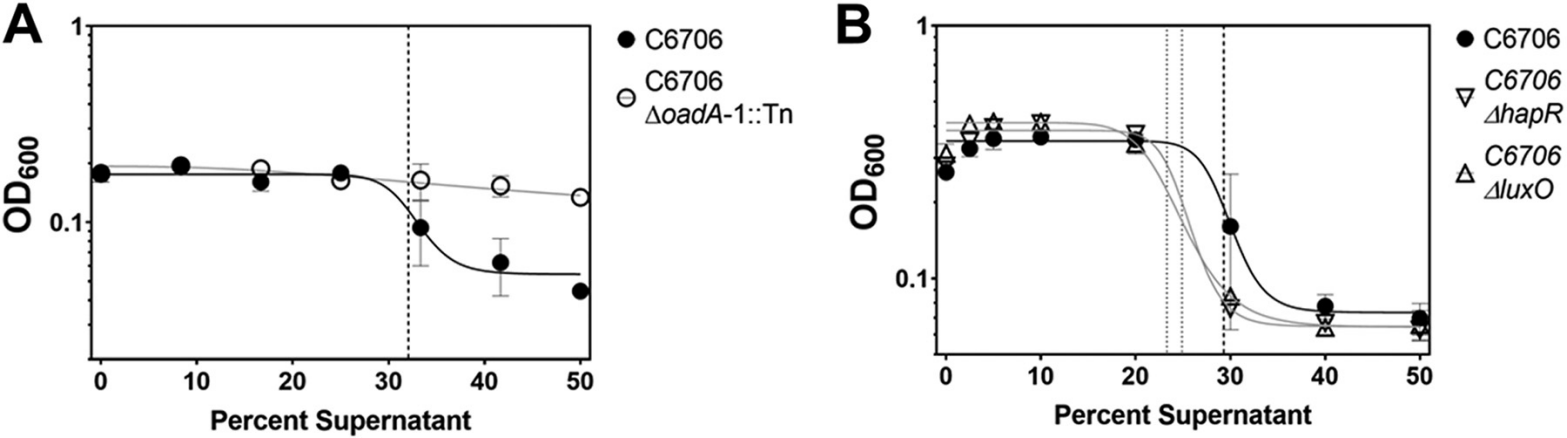
Quantification of the V. cholerae supernatant inhibitory activity of S. flexneri using a cell-free assay. (A) Disruption of a V. cholerae oxaloacetate decarboxylase (*oadA-1*) eliminates supernatant inhibitory activity. Supernatants derived from the growth of the V. cholerae Δ*oadA*-1::Tn mutant were unable to inhibit S. flexneri CFS100 growth, compared with the W.T. strain. The IC_50_ of the W.T. strain is shown by the dashed line. (B) Disruption of V. cholerae quorum sensing master regulators does not significantly alter supernatant inhibition activity. Supernatants derived from the growth of V. cholerae Δ*hapR*::Tn or Δ*luxO*::Tn mutants were able to inhibit the growth of S. flexneri CFS100 similar to the V. cholerae C6706 W.T. strain. Each point is the mean of 3 replicates, and error bars show standard deviation. Trendlines show a nonlinear regression (log inhibitor versus response, 4 parameter). IC_50_ values are shown by the dashed (W.T.) and dotted (Δ*hapR* and Δ*luxO*) lines.

Very few studies have experimentally examined the basis of V. cholerae citrate metabolism (15). V. cholerae C6706 encodes genes associated with the citric acid cycle (TCA) and also citrate fermentation. In this pathway, a sodium-citrate symporter facilitates citrate uptake and then a citrate lyase (ACLY) cleaves citrate into acetate and oxaloacetate, of which oxaloacetate is converted to CO_2_ and pyruvate by oxaloacetate decarboxylase (33–36) (Fig. 3). To determine if citrate fermentation contributes to enteric growth inhibition, we tested two V. cholerae C6706 transposon mutants with disrupted genes encoding ACLY, namely, Δ*citE*::Tn and Δ*citF*::Tn, to determine the importance of the citrate metabolism pathway on the V. cholerae C6706 competition phenotype (37). Although both of these mutants still displayed growth on Simmons’ citrate agar, they did not prevent E. coli growth (Table 1). Similarly, a mutation in *oadA-1*, coding for oxaloacetate decarboxylase, which blocks the conversion of oxaloacetate to pyruvate in citrate metabolism (34), also eliminated the inhibitory activity produced by V. cholerae C6706 in a supernatant inhibition assay (Fig. 4A). We concluded that the inhibitory activity of V. cholerae C6706 on S. flexneri and E. coli is dependent on V. cholerae C6706 citrate fermentation.

### V. cholerae C6706 raises the pH of media during growth.

We sought to characterize the nature of the V. cholerae C6706 inhibition of S. flexneri. The secretion of inhibitory factors is a bacterial behavior that often conveys a communal fitness advantage (38, 39); therefore, we hypothesized that the V. cholerae inhibitory mechanism was regulated by quorum sensing. However, we found that there was no significant difference in the IC_50_ of wild-type (W.T.) V. cholerae C6706 supernatants (Fig. 4B, 29.3, dashed line) compared with that of Δ*hapR* (Fig. 4B, 24.9 dotted line) and Δ*luxO* (Fig. 4B, 23.3, dotted line) quorum-sensing mutant strains in our supernatant inhibition assay (Fig. 4B). We also investigated the hypothesis that the V. cholerae C6706 secreted factor is a protein (28, 30). If the V. cholerae C6706 secreted factor was a protein, it might be sensitive to heat; however, V. cholerae C6706 cell-free supernatants that were incubated at 60°C for 60 min still retained their ability to inhibit S. flexneri growth (Table 2). Furthermore, proteinase K treatment of V. cholerae C6706 cell-free supernatants also was not able to significantly alleviate S. flexneri growth inhibition, as well as filtration through a 1-kDa filter (Table 2). These data together do not support the hypothesis that the V. cholerae C6706 secreted inhibitory factor is a protein.

**TABLE 2:** Supernatant treatment effects on the ability to inhibit S. flexneri CFS100 growth^*a*^

Supernatant producing strain	Supernatant inhibition assay results				
	No treatment	Heat	Proteinase K	Filtration	Methanol extraction
V. cholerae C6706	+	+	+	+	−
S. flexneri CFS100	−	−	−	−	−

a V. cholerae C6706 or S. flexneri CFS100 were grown for 72 h in LB, cells were removed, and then cell-free supernatants were treated and used in a supernatant inhibition assay to inhibit the growth of S. flexneri C6706. Treatments included heat treatment (60°C for 1 h), treatment with proteinase K, filtration through a 1-kDa filter, or methanol phase separation followed by reconstitution in saline. +, indicates supernatants were able to inhibit the growth of S. flexneri CFS100; −, indicates that supernatants were not able to inhibit the growth of S. flexneri CFS100.

We next investigated the hypothesis that the V. cholerae C6706 secreted inhibitory factor is a metabolic by-product (29). We performed a methanol extraction to isolate metabolites from V. cholerae C6706 cell-free supernatants. Methanol was added to V. cholerae C6706 cell-free supernatants at a ratio of 6:1 to precipitate protein. The liquid fraction was collected and evaporated by vacuum centrifugation; the remaining content was resuspended in saline. We found that metabolites extracted from V. cholerae C6706 cell-free supernatants did not display any inhibitory activity toward S. flexneri (Table 2).

As part of this experiment, we measured the pH of the supernatants before and after extraction and measured the pH of metabolites resuspended in saline after methanol extraction. Prior to culture, the pH of the LB used to grow V. cholerae C6706 and S. flexneri was approximately 7.0; however, the pH of V. cholerae C6706 cell-free supernatants after 48 h of culture was approximately 9.5, consistent with prior studies (29). After methanol extraction, the pH of V. cholerae C6706 cell-free supernatant metabolites resuspended in saline was approximately 7.0. We also measured the pH of CB before and after V. cholerae C6706 growth. Like LB, the pH of liquid CB was approximately 7.0 before inoculation; following 48-h growth of C6706 in CB, the pH rose by approximately two logs to 9.0. To determine if other V. cholerae strains raise medium pH similar to the C6706 strain, we grew each of our V. cholerae strains in LB for 48 h and then quantified the pH of cell-free supernatants. Only the V. cholerae C6706 and DL4211 strains demonstrated a pH of >9; notably, there was variation in the medium pH of several V. cholerae strains after 48 h of growth (Table 1).

### V. cholerae C6706 alkalization of media inhibits S. flexneri growth.

**FIG 5:**
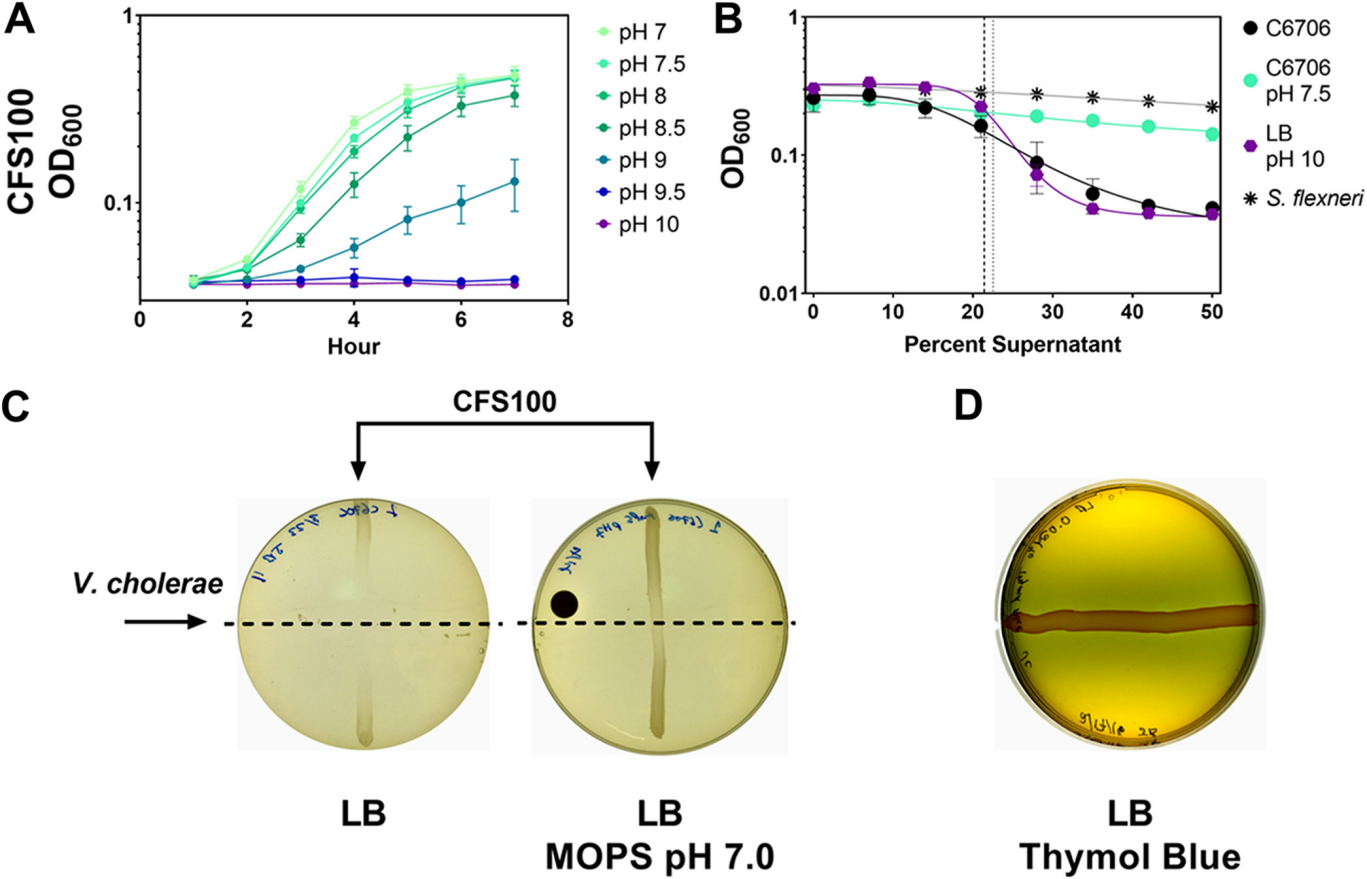
Media alkalization is necessary for V. cholerae supernatant inhibitory activity. (A) Alkaline pH inhibits S. flexneri CFS100 growth. LB was adjusted to different pHs using sodium hydroxide, and S. flexneri growth was quantified over time by measuring OD_600_. When the media pH exceeded 9.0, significant S. flexneri growth inhibition occurred. (B) Changes in pH correspond to V. cholerae supernatant inhibitory activity. The supernatant inhibition assay was used to quantify the inhibitory activity of V. cholerae supernatants with adjusted pH. When the pH of V. cholerae was lowered to 7.5, no S. flexneri growth inhibition was observed; in contrast, when the pH of LB was raised to 10.0, the pattern of growth inhibition was similar to that of V. cholerae supernatants. (C) Buffering LB agar plates reduces V. cholerae supernatant inhibitory activity. LB agar was prepared with and without MOPS buffer (pH 7.0; 50 mM). When the cross-streak assay was performed, there was a minimal zone of inhibition observed in the MOPS plate, compared with the LB agar plate. (D) Thymol blue was supplemented to LB agar to visualize pH changes after V. cholerae growth. After 24 h, a blue coloration change is observed surrounding the V. cholerae streak corresponding with the zone of inhibition we observe, indicating an increase in medium pH.

V. cholerae can grow in pHs between 6.5 and 9, with an optimal growth pH of 8 (40, 41); E. coli and S. flexneri do not grow when the external pH is higher than 9.0 (42). To confirm that S. flexneri is also sensitive to basic conditions, we grew S. flexneri in LB adjusted to different pHs. We observed a modest growth defect when the pH of LB was brought to 9.0, and we observed no growth when the pH was raised to 9.5 and 10.0 (Fig. 5A). Because the pH of V. cholerae C6706 supernatants was higher than 9.0 after 72 h, we hypothesized that alkaline pH contributed to the inhibition of S. flexneri growth. We tested this hypothesis by adjusting the pH of V. cholerae C6706 cell-free supernatants. When the pH of V. cholerae C6706 cell-free supernatants was adjusted to 7.5 using HCl, we found that V. cholerae C6706 cell-free supernatants could not inhibit S. flexneri growth in a liquid inhibition assay (Fig. 5B).

Furthermore, when the pH of LB was raised to 10.0, it had the same inhibitory effect toward S. flexneri as V. cholerae C6706 supernatants (Fig. 5B). To determine if this same effect occurred in our cross-streaking assay, we grew V. cholerae C6706 on LB agar buffered with morpholinepropanesulfonic acid (MOPS; 50 mM) at pH 7.0. When we performed our cross-streaking assay using this buffered medium, we observed little to no inhibition of S. flexneri growth (Fig. 5C). We also visualized V. cholerae C6706 alkalization of LB agar by adding thymol blue, a pH indicator that transitions from yellow to blue between pH 8.0 and 9.2. After 24 h, we observed a blue zone surrounding V. cholerae C6706 growth, which corresponded to the change in pH we observed in liquid medium (Table 1) and the S. flexneri zone of inhibition we observed (Fig. 5D).

**FIG 6:**
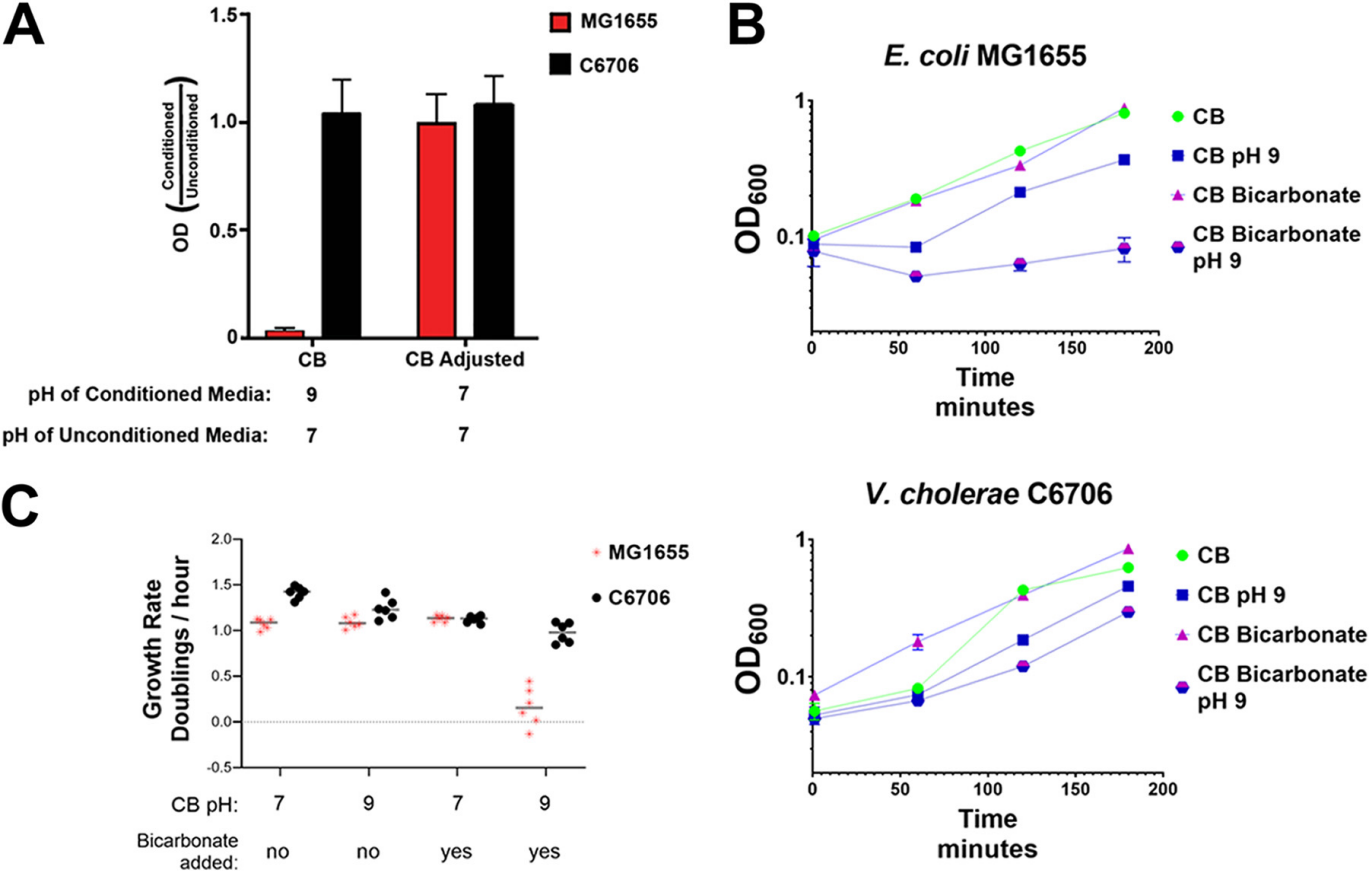
The growth inhibition of E. coli can be replicated in a *Vibrio*-free system using a high pH and bicarbonate. (A) Conditioned CB medium by growth of V. cholerae prevents the growth of E. coli in a pH-dependent manner. Shown is the optical density of indicated bacterial culture after 4 h of growth in different conditioned media compared with that of unconditioned media. V. cholerae C6706 was grown in CB media for 48 h. The pH cell-free supernatant was then either adjusted to 7.0 or left as indicated. The relative ability of both E. coli and V. cholerae to grow in this conditioned media was determined by the optical density after 4 h compared with the optical density in unconditioned media. (B) Preventing the growth of E. coli can be mimicked with pH and bicarbonate. CB medium is either supplemented with bicarbonate or not and either left at pH 7 or artificially raised to a pH of 9. Top, shows the growth of E. coli MG1655 in different media conditions; bottom, shows the growth of V. cholerae C6706. (C) Growth rates of both E. coli and V. cholerae were determined in these four media by taking the slope of the linear portion of the growth curve. The *y* axis represents the growth rate of E. coli divided by the growth rate of V. cholerae under the conditions indicated on the *x* axis.

Similar to growth in LB, when V. cholerae C6706 was grown in CB, we found that V. cholerae C6706 cell-free supernatants did not inhibit E. coli growth after the pH was readjusted to ~7.0 with HCl or if the V. cholerae C6706 growth medium was buffered to prevent the increase in pH (Fig. 6A). Carbonate is a by-product of citrate metabolism under basic conditions and is known to prevent the growth of E. coli (43), and thus, we hypothesized that V. cholerae C6706 metabolism of citrate generates carbonates and basic conditions responsible for deprotonating bicarbonate to carbonate (Fig. 3). To test the hypothesis, we examined E. coli and V. cholerae C6706 growth in a combination of sodium bicarbonate and a basic pH, mimicking the conditions we anticipate are caused by V. cholerae C6706 grown in citrate-containing media. We found that these conditions restricted the growth of E. coli more profoundly than basic pH or bicarbonate alone (Fig. 6B). V. cholerae C6706 grew under all conditions, and even though V. cholerae C6706 grew more slowly in the combination of high pH and bicarbonate (Fig. 6B), it still reached the mid-logarithmic phase of growth. The supplementation of bicarbonate to media at a pH of 9.0 reduced the growth rate of E. coli but not that of V. cholerae (Fig. 6C).

## DISCUSSION

It was first reported some 50 years ago that some V. cholerae strains inhibit the growth of enteric bacteria, but the mechanism of this inhibition was not determined (28–30). While more recent studies have illustrated how V. cholerae kills other bacteria using a type VI secretion system (T6SS) (3), the mechanism first described by Chakrabarty et al. (28) is a contact-independent mechanism. Here, we investigated the hypotheses originally posited by these groups regarding the V. cholerae mechanism of contact-independent inhibition of enteric growth *in vitro* (28–30). Our findings were consistent with the findings of Bhaskaran et al. (29), who proposed that secreted carbonates raised media pH and caused enteric growth inhibition. We provide additional support of this hypothesis by demonstrating that V. cholerae C6706 mutants defective in the conversion of citrate to oxaloacetate (*citE* and *citF*) and the conversion of oxaloacetate to pyruvate (*oadA-1*) were not able to inhibit enteric growth (Table 1; Fig. 4A). There are limitations to this study that are worth noting. These experiments are artificial in nature, and they show only the effects of V. cholerae secreted products after extended growth. All experiments were performed in lab medium, which is very different from a marine or host intestinal environment, and so we cannot ascertain the significance of enteric inhibition during host colonization. We cannot rule out the possibility that V. cholerae C6706 produces a bacteriocin-like protein to inhibit the growth of enteric bacteria; however, our data suggest that such a protein would require an alkaline environment to function, as buffering media pH or readjusting V. cholerae C6706 cell-free supernatants to a neutral pH abolishes inhibitory activity (Fig. 5 and 6).

Carbohydrate metabolism is an essential aspect of V. cholerae pathogenesis. Previous studies have shown that V. cholerae relies on standard carbon metabolism pathways coupled with oxygen respiration in the host, including the Embden-Meyerhof-Parnas (glycolysis) and Entner-Doudoroff pathways (44–46). Recent studies demonstrate that pyruvate dehydrogenase and pyruvate formate lyase, enzymes that facilitate the transition to TCA or fermentation by converting pyruvate to acetyl-coenzyme A (CoA), are essential for V. cholerae pathogenesis in an infant mouse model (47) and that citrate metabolism intersects this metabolic node. The citrate metabolic axis in particular is a defining aspect of V. cholerae, which is commonly used to differentiate V. cholerae from other bacteria (13). Citrate metabolism genes are highly conserved in V. cholerae (14), and citrate fermentation promotes V. cholerae pathogenesis in an infant mouse model (15).

Notably, V. cholerae citrate fermentation, along with lactate and acetate metabolism, produce carbonates as a by-product (29). V. cholerae C6706 also encodes at least three putative carbonic anhydrases (VC0586, VCA0274, and VC0058) that potentially contribute to the accumulation of environmental carbonates (48, 49). Carbonates incorporate free H+ ions to produce CO_2_, which raises pH. Additionally, OadA-1 is a decarboxylase that consumes a proton in the conversion of oxaloacetate to pyruvate, which could also contribute to environmental alkalization (34). The human host also secretes bicarbonate into the intestine (50), and bicarbonate serves as an important signal in V. cholerae pathogenesis. Bicarbonate is a critical component of AKI conditions to induce cholera toxin production *in vitro* (51). It has also had more recently been shown that bicarbonate regulates the expression of the virulence regulator *toxT* and the levels of the second messenger cyclic di-GMP (18, 52, 53). V. cholerae has a high tolerance for carbonates, and carbonates also inhibit the growth of E. coli at alkaline pH (43). We demonstrate here that high pH and carbonates have a synergistic effect at inhibiting E. coli growth (Fig. 6). We postulate that mutations in the citrate fermentation pathway reduce carbonate production enough to abrogate enteric growth inhibition.

Chakrabarty et al. (28) first observed that V. cholerae inhibits the growth of E. coli when grown on citrate-containing media, but we have demonstrated that external citrate is not required for this behavior (Fig. 1). This result is consistent with previous findings, where V. cholerae was able to inhibit enteric growth on acetate and lactate medium (29). It is surprising that the V. cholerae C6706 Δ*citE* and Δ*citF* mutants were able to grow on Simmons’ citrate agar (Table 1). Despite its widespread prevalence in *Vibrio* spp., little is known about V. cholerae citrate metabolism (15). In these mutant strains, citrate could be converted to oxaloacetate via the glyoxylate shunt, feeding into gluconeogenesis for the production of biomass. This pathway is mediated by isocitrate lyase (ICL), which V. cholerae C6706 carries (VC0736). Consistent with this alternate citrate metabolic pathway, we simulated V. cholerae metabolism using a previously constructed genome-scale metabolic model based on the V52 strain (54) with the software OptFlux (55). We found that disruption of ACLY did not impact V. cholerae growth *in silico* when citrate was the sole carbon source, with both producing equal biomass values (0.56). This process was dependent on the glyoxylate shunt, as disruption of both ACLY and ICL resulted in no biomass production *in silico*. Conversely, several of the V. cholerae strains examined in this study were not capable of citrate fermentation on Simmons’ agar. Currently, there are not annotated genomic sequences for all of these *Vibrio* strains, but at least one strain (MZO-2) carries citrate lyase genes with >99% protein identity to the N16961 strain, and yet, it does not grow on Simmons’ agar, suggesting that there may be differential expression of citrate metabolism genes among these strains. There are also examples of V. cholerae acquiring mutations in conserved metabolic pathways (56).

Citrate metabolism is not necessarily indicative that a V. cholerae strain inhibits enteric growth, suggesting other systems work in conjunction with citrate metabolism to create a hostile growth environment for E. coli and S. flexneri (Table 1). While we demonstrate that the citrate metabolic axis is required for V. cholerae C6706 inhibition of enteric growth, it is likely that other secreted molecules also contribute to this process, as we did not observe a consistent correlation among V. cholerae strains between growth on citrate, the pH of LB media after 48 h, and enteric growth inhibition (Table 1). This finding suggests that there are multiple ways in which different *Vibrio* strains can inhibit enteric growth. This hypothesis is similar to those made in previous studies, where they observe different patterns of V. cholerae enteric growth inhibition (28, 30). Notably, C6706 and N16961 are genetically similar (57, 58) and yet produce different pH after extended growth (Table 1); there is evidence that lab domestication has impacted some phenotypes of these strains, which could include enteric inhibition (59, 60). There is diversity among *Vibrio* strains in their metabolic pathways; for example, some V. cholerae El Tor biotypes have developed neutral fermentation pathways, resulting in 2,3-Butanediol production, to avoid creating potentially harmful organic acids (25, 26). It is possible that certain V. cholerae strains produce other secondary metabolites that also contribute to enteric growth inhibition, independent of citrate fermentation. One possibility is the production of polyamines, such as cadaverine which some V. cholerae species produce in high abundance (61, 62). The synthesis of cadaverine consumes protons and protects V. cholerae from acid stress, and also inhibits enteric bacteria at high pH (63). Further studies will reveal how other secondary metabolites contribute to V. cholerae inhibition of enteric bacterial growth.

This report describes an example of how V. cholerae C6706 uses metabolic products to outcompete other bacteria *in vitro*. Specifically, we propose that creating a highly alkaline environment is a mechanism for V. cholerae C6706 to generate a niche. The concept of V. cholerae using secreted metabolites to protect its niche is not entirely novel, as a previous study has shown that V. cholerae releases ammonium when grown on chitin to inhibit protist grazing (64). Creating a niche diminished of other species of bacteria would allow V. cholerae to suppress competing commensal bacteria and control its nutrient pool. In combination with other known systems, such as the T6SS, this mechanism of competition likely contributes to the survivability, adaptability, and success of V. cholerae as a pathogen and prominent aquatic bacterium.

## MATERIALS AND METHODS

### Strains and culture conditions.

Strains included in this study are E. coli MG1655 and S. flexneri 2457T (CFS100 [65]), as well as V. cholerae strains listed in Table S1 in the supplemental material. V. cholerae and E. coli strains were grown in lysogeny broth (LB) (1% tryptone, 0.5% yeast extract, and 0.5% NaCl) at 37°C with shaking. As necessary, bacteria were grown in the presence of 50 μg/mL kanamycin, 100 μg/mL streptomycin, or 50 μ g/mL rifampicin. Cross streaking and conditioned medium assays were performed using LB or citrate media broth (CB). Briefly, nutrient broth no. 2 (Oxoid) was supplemented with NH_4_Cl (0.03%), K_2_HPO_4_ (0.5%), and sodium citrate (0.5%) and includes EGTA (3.0 mg/mL).

To determine whether a bacterium could utilize citrate as a sole carbon source, strains were grown on Simmons’ citrate agar. Ammonium dihydrogen phosphate (0.2 g/L), disodium ammonium phosphate (0.8 g/L), magnesium sulfate heptahydrate (0.2 g/L), and sodium chloride (5.0 g/L) salts were added to a solution of 2.0 g/L trisodium citrate, with 1.5% (wt/vol) agar.

### Cross-streaking assay.

The cross-streaking assay was performed as described previously (66). Briefly, liquid from an overnight culture of the producing bacteria was streaked down the center of an agar plate (LB or CB) using a sterile cotton-tipped stick, resulting in a ~3/4″ wide stripe and was left to grow for 48 h at 37°C followed by 6 h at 4°C. The resulting growth was manually removed by scraping the plate with a sterile cotton-tipped stick, and the remaining bacteria were killed by exposure to chloroform vapor for 30 min. Residual chloroform was allowed to evaporate from the plate for 30 min in the fume hood. Afterward, the indicator strain (for example, E. coli or S. flexneri CFS100) was streaked in a line perpendicular to the producing bacteria from an overnight culture in the same manner. The plate was then incubated for 18 h at 37°C. For visualizing pH changes in the agar plate, an LB agar plate with 0.0032% (wt/vol) thymol blue was inoculated with a single streak with V. cholerae C6706 from an overnight culture and incubated statically for 24 h at 37°C.

### Conditioned medium assay.

The concentration of our LB medium prior to bacterial growth was approximately 7.0. Both V. cholerae and E. coli or S. flexneri CFS100 were grown for 48 h at 37°C. The resulting culture was centrifuged, the supernatant was retained, and the supernatant was filter sterilized using 0.22-μm Millipore polyvinylidene difluoride (PVDF) filters. The V. cholerae supernatant (either diluted with saline or not) or medium at a specific pH was mixed 1:1 with a 1:1,000 dilution of an S. flexneri CFS100 overnight culture in LB to a total volume of 100 μL in each well of a 96-well plate. The V. cholerae supernatant produced in LB was used to perform the inhibition assays, using supernatant dilutions ranging from 50% to 0% (final concentration). Plates were then incubated at 37°C, shaking at 200 rpm. OD_595_ was measured at the 6-h time point using an Opsys MR plate reader.

LB was adjusted to various pHs with NaOH and autoclaved. Supernatant from V. cholerae grown in neutral LB was adjusted to multiple pHs with 1 M NaOH or 1 M HCl, as appropriate, and was filter sterilized using an 0.22-μm polyethersulfone (PES) membrane filter. The V. cholerae supernatant and LB at close pHs were combined 1:1, and their pHs were redetermined. The resulting media (either LB or 50% V. cholerae supernatant) were then used to perform growth inhibition assays as described above. Media were mixed 20:1 with a 1:100 inoculum of a S. flexneri CFS100 overnight culture. OD_595_ was measured at the 6-h time point.

To determine if supernatants contained an inhibitory protein, sterile supernatants were prepared as described previously, and then these supernatants were either filtered through a 1-kDa nominal molecular weight (NMW) ultrafiltration disc (Millipore) or treated with proteinase K. Briefly, proteinase K powder was dissolved at a concentration of 20 mg/mL in sterile 50 mM Tris (pH 8.0) and 1.5 mM calcium acetate (cite proteinase K recipe). A total of 20 μL of this solution was added to 2 mL of supernatant and incubated at 50°C for 20 min. The supernatant was then incubated at 70°C for 10 min to inactivate the proteinase K, before it was used in our conditioned medium assay.

Methanol extraction of metabolites was performed as described previously, and extractions were performed in triplicate (67). Sterile supernatants were prepared as previously described above and then 600 μL cold MeOH was added to the 100 μL V. cholerae supernatant. The mixture was vortexed and then centrifuged for 2 min at maximum (max) speed at 4°C. A total of 100 μL chloroform was then added to the mixture, then vortexed, and centrifuged for 2 min at max speed at 4°C. A total of 300 μL water was then added to the mixture, which was then vortexed and centrifuged for 2 min at max speed at 4°C. The aqueous phase was then transferred to a new tube, and 300 μL MeOH was added. The mixture was evaporated by vacuum centrifugation, and the remaining material was resuspended in 100 μL sterile saline.

### Growth assays.

For growth curves of S. flexneri in different concentrations of the V. cholerae C6706 supernatant, overnight cultures of S. flexneri CFS100 were diluted (1:100) into 96-well plates containing LB with decreasing concentrations of the cell-free supernatant; plates were incubated at 37°C with shaking, and the optical density at 600 nm of bacterial cultures was measured and recorded every 20 min for 10 h using a BioTek Synergy H1 plate reader. Data were analyzed using the R package grofit (68), with the Richards growth model.

For growth curves of S. flexneri in different pH media, overnight cultures of S. flexneri CFS100 were diluted (1:100) into LB at different pHs, and the optical density at 600 nm of bacterial cultures was measured and recorded every hour for 7 h. For comparing the relative growth rates of E. coli and V. cholerae, overnight cultures of V. cholerae or E. coli were diluted (1:100) into CB in the presence or absence of bicarbonate buffered to pH of 7 or 9. OD_600_ readings were recorded every hour. The resulting ODs were plotted on a graph versus time, and the portion of the graph that was linear was used to calculate a slope. The slope of E. coli was then divided by the slope of V. cholerae grown under the same growth conditions.

### *In silico* analysis of V. cholerae metabolism.

*In silico* metabolism simulations were performed using a previously published V. cholerae genome-scale metabolic network reconstruction (54) and OptFlux (55). Although V. cholerae carries a sodium/citrate symporter (VC0795), there was no reaction for citrate transport in this model (54), so citrate exchange was modified to be cytoplasmic. We removed external boundary metabolites and used the core biomass production as our objective function and the parsimonious Flux-Balance Analysis simulation method. To simulate growth in Simmons’ media, default environmental conditions were used, except that the lower bound of glucose exchange was set to 0 and the lower bound of citrate exchange was set to −20. Total biomass production was limited by carbon availability under these simulated conditions.

### Statistical analysis.

All statistical analysis was performed using GraphPad Prism. For supernatant inhibition assays, data were regressed using a nonlinear four-parameter variable slope inhibitor model, and IC_50_ values were compared using a sum-of-squares F-test (*P* < 0.05). For growth analysis, data were compared using analysis of variance (ANOVA) (*P* < 0.05).

### Data availability.

All data generated or analyzed during this study are included in this published article and in File S1 in the supplemental material.

## Supplementary Material

Reviewer comments
