# Supplementary material for: Vibrio cholerae Alkalizes Its Environment via Citrate Metabolism to Inhibit Enteric Growth In Vitro
Source: Microbiol Spectr. 2023 Mar 14;11(2):e04917-22. doi: 10.1128/spectrum.04917-22 (PMC10100763; doi:10.1128/spectrum.04917-22)
Supplement: Supplemental file 1 — Table S1. Download spectrum.04917-22-s0001.pdf, PDF file, 0.1 MB [file spectrum.04917-22-s0001.pdf]

**Table S1**. Strains used in this study.

| Strain Name | Serotype | Biotype | Cholera toxin | Source | Reference |
|---|---|---|---|---|---|
| C6706 | 01 | El Tor | + | Peru, 1961 | PMC174155 |
| C6706 Δ*citE*::Tn | 01 | El Tor | + | | PMC2438431 |
| C6706 Δ*citF*::Tn | 01 | El Tor | + | | PMC2438431 |
| C6706 Δ*oadA-1*::Tn | 01 | El Tor | + | | PMC2438431 |
| N16961 | 01 | El Tor | + | Bangladesh, 1975 | 10.1038/35020000 |
| O395 | 01 | Classical | + | India, 1965 | John Mekalanos (Harvard Medical School, Boston, MA, USA) |
| DL4211 | 0123 | | - | USA, 2008 | PMC3482179 |
| 1587 | 012 | | - | Peru, 1994 | PMC2741270 |
| V52 | 0137 | | + | Sudan, 1968 | PMID: 4564589 |
| MZO-3 | 0139 | | - | Bangladesh, 2001 | Michelle Dziejman (University of Rochester, New York) |
| 2740-80 | 01 | El Tor | - | USA, 1980 | Jun (Jay) Zu (University of Pennsylvania) |
| MAK-757 | 01 | El Tor | + | Indonesia, 1937 | Michelle Dziejman (University of Rochester, New York) |
| AM19226 | 039 | | - | Bangladesh, 2001 | Michelle Dziejman (University of Rochester, New York) |
| V51 | 0141 | | | USA, 1987 | Michelle Dziejman (University of Rochester, New York) |
| NIH41 | 01 | Classical | + | | John Mekalanos (Harvard Medical School, Boston, MA, USA) |
| MZ02 | 014 | | - | Bangladesh, 2001 | Jun (Jay) Zu (University of Pennsylvania) |
| C6709 | 01 | El Tor | | Peru, 1991 | John Mekalanos (Harvard Medical School, Boston, MA, USA) |
| CA401 | 01 | Classical | + | India, 1953 | PMC550806 |
